# Interspecies Single‐Cell RNA‐Seq Analysis Reveals the Novel Trajectory of Osteoclast Differentiation and Therapeutic Targets

**DOI:** 10.1002/jbm4.10631

**Published:** 2022-05-16

**Authors:** Yasunori Omata, Hiroyuki Okada, Steffen Uebe, Naohiro Izawa, Arif B. Ekici, Kerstin Sarter, Taku Saito, Georg Schett, Sakae Tanaka, Mario M. Zaiss

**Affiliations:** ^1^ Department of Internal Medicine 3, Rheumatology and Immunology Friedrich‐Alexander‐University Erlangen‐Nürnberg (FAU) and Universitätsklinikum Erlangen Erlangen Germany; ^2^ Department of Orthopaedic Surgery, Faculty of Medicine The University of Tokyo Tokyo Japan; ^3^ Bone and Cartilage Regenerative Medicine, Graduate School of Medicine The University of Tokyo Tokyo Japan; ^4^ Center for Disease Biology and Integrative Medicine The University of Tokyo Tokyo Japan; ^5^ Institute of Human Genetics University of Hospital, Friedrich‐Alexander‐University Erlangen‐Nürnberg (FAU) Erlangen Germany; ^6^ Department of Orthopaedic Surgery Saitama Medical University Saitama Japan

**Keywords:** ChIP‐SEQUENCING, HISTONE MODIFICATION, OSTEOCLAST, RAB38, SINGLE‐CELL RNA‐SEQUENCING

## Abstract

Bone turnover is finely tuned by cells in the bone milieu, including osteoblasts, osteoclasts, and osteocytes. Osteoclasts are multinucleated giant cells with a bone‐resorbing function that play a critical role in regulating skeletal homeostasis. Osteoclast differentiation is characterized by dramatic changes in morphology and gene expression following receptor activator of nuclear factor‐kappa‐Β ligand (RANKL) stimulation. We performed single‐cell RNA‐sequencing analyses of human and murine osteoclast‐lineage cells (OLCs) and found that OLCs in the mitotic phase do not differentiate into mature osteoclasts. We also identified a guanosine triphosphatase (GTPase) family member, RAB38, as a highly expressed molecule in both human and murine osteoclast clusters; *RAB38* gene expression is associated with dynamic changes in histone modification and transcriptional regulation. Silencing *Rab38* expression by using short hairpin RNA (shRNA) inhibited osteoclast differentiation and maturation. In summary, we established an integrated fate map of human and murine osteoclastogenesis; this will help identify therapeutic targets in bone diseases. © 2022 The Authors. *JBMR Plus* published by Wiley Periodicals LLC on behalf of American Society for Bone and Mineral Research.

## Introduction

Skeletal homeostasis is maintained by the sequential process of bone resorption and bone formation, known as bone remodeling,^(^ ^1^, ^2^ ^)^ which controls bone density and quality. Disruption of this process can result in bone disorders such as osteoporosis and osteopetrosis. Osteoclasts are multinucleated giant cells that primarily regulate both physiologic and pathologic bone resorption. Osteoclasts differentiate from monocyte–macrophage lineage osteoclast precursors (OCPs) in response to two critical cytokines, receptor activator of nuclear factor‐kappa‐B ligand (RANKL) and macrophage colony‐stimulating factor (M‐CSF). Upon the binding of RANKL to its receptor, RANK, expressed on OCPs, downstream intracellular signaling pathways mediated by tumor necrosis factor receptor‐associated factor 6 (TRAF6) are activated, and critical transcription factors, such as nuclear factor of activated T‐cells (NFAT) c1, c‐Fos, and nuclear factor (NF)‐κB are induced. Additionally, co‐stimulatory signaling, mediated by DAP12 and Fc receptors, plays an essential role in osteoclast differentiation. We recently reported the transcriptional landscape during osteoclast differentiation^(^ ^3^ ^)^ and demonstrated that the Spi‐1 proto‐oncogne transcription factor switches binding partners from interferon regulatory factor 8 (IRF8) to NFATc1, which is associated with changes in epigenetic profiles and induction of cell‐type‐specific gene expression.

Despite general similarities between human and murine osteoclast differentiation, several species‐related differences exist. For example, although M‐CSF is essential for both human and murine osteoclast differentiation,^(^ ^4^, ^5^ ^)^ M‐CSF stimulates proliferation of murine OCPs but not human OCPs. In addition, longer stimulation periods are required for human osteoclastogenesis than for murine osteoclastogenesis. Transforming growth factor (TGF)‐β is indispensable for murine osteoclast differentiation,^(^ ^6^ ^)^ but exerts both stimulatory and inhibitory effects on human osteoclastogenesis.^(^ ^7^ ^)^ Both human and murine regulatory T cells inhibit differentiation of osteoclasts and arthritic bone destruction,^(^ ^8^, ^9^, ^10^ ^)^ and activated T cells strongly inhibit murine osteoclast differentiation. However, activated T cells in humans support osteoclast formation via RANKL‐dependent and RANKL‐independent mechanisms, and they are also involved in osteoclast‐mediated bone destruction in human inflammatory arthritis.^(^ ^11^, ^12^, ^13^ ^)^ Such differences in osteoclast differentiation processes between humans and mice make it difficult to directly extrapolate murine data to humans. Therefore, understanding in further detail the similarities and differences underlying human and murine osteoclast differentiation processes is critical for identifying therapeutic targets for osteoclast‐related diseases.

In this study, we performed single‐cell RNA‐sequencing (scRNA‐seq) analysis in both human and murine in vitro osteoclast cultures to identify subgroups of osteoclast lineage cells (OLCs) involved in osteoclast differentiation and addressed the similarities and differences between human and murine OLCs. By integrating single‐cell data from different species, we identified genes explicitly induced during osteoclast differentiation in both human and murine cultures. Recently, several reports have been published that analyzed the osteoclast differentiation process in mice at the single cell level,^(^ ^14^, ^15^, ^16^ ^)^ but our study is the first to integrate data sets across species to clarify the similarities and differences between human and murine osteoclast differentiation processes. Through these approaches, we have identified a possible therapeutic target, RAB38, which plays a critical role in osteoclast differentiation and activation.

## Materials and Methods

### Human and murine osteoclast differentiation

All experiments using human and murine materials were approved by the local ethics committees of University Erlangen and The University of Tokyo. Informed consent was obtained from a healthy volunteer to collect blood samples. After diluting peripheral blood 1:1 with phosphate‐buffered saline (PBS; Gibco, Grand Island, NY, USA), peripheral blood mononuclear cells (PBMCs) were collected by the density gradient centrifugation method using Lymphoflot (Bio‐Rad Laboratories, Hercules, CA, USA) (diluted blood: Lymphoflot = 2:1). Human PBMCs were cultured with 30 ng/mL recombinant human M‐CSF (PeproTech, Rocky Hill, NJ, USA; 300‐25) and 100 ng/mL recombinant human RANKL (Pepro Tech, Rocky Hill, NJ, USA; 310‐01) in α modified essential medium (α‐MEM; Gibco, Grand Island, NY, USA) supplemented with 10% heated‐inactivated fetal calf serum (FCS; Gibco) and 1% penicillin–streptomycin (Gibco) to differentiate into osteoclasts.

Murine bone marrow cells were isolated from 8‐week‐old to 10‐week‐old C57BL/6J mice (Charles River Laboratories, Erkrath, Germany; Sankyo Labo Service Corporation, Tokyo, Japan), by flushing the femur and tibial bones. After culturing the cells overnight in α‐MEM with 10% heat‐inactivated FCS and 1% penicillin–streptomycin (Gibco), in vitro cultured murine osteoclasts were differentiated using 10 ng/mL recombinant murine M‐CSF (R&D Systems, Minneapolis, MN, USA) and 100 ng/mL recombinant murine RANKL (PeproTech). All mice used in this study were housed under specific pathogen‐free conditions. The mice received water and feed ad libitum. The animal rooms kept the temperature at 22°C to 23°C and a humidity of 50% to 60%. The rooms maintain a 12‐hour light‐dark rhythm. Animals are kept in type II long cages, with a maximum of five animals. The protocols for animal experiments were approved by the local ethics authorities in University Erlangen and The University of Tokyo.

After culturing osteoclasts, the cells were stained using a tartrate‐resistant acid phosphatase (TRAP) staining kit (Sigma‐Aldrich, St. Louis, MO, USA). TRAP‐positive cells with more than two nuclei were considered to be mature osteoclasts.

### Single‐cell RNA‐sequencing preparation

In vitro cultured human and murine osteoclasts after 14‐day and 4‐day RANKL stimulation, respectively, were subjected to 10× Chromium Single‐Cell 3′ Solution v2 library preparation (10x Genomics, Pleasanton, CA, USA), according to the manufacturer's instructions. Library sequencing was performed on an Illumina HiSeq 2500 sequencer (Illumina, San Diego, CA, USA) to a depth of 100 million reads each.

### Bioinformatics analysis of single‐cell RNA‐sequencing

Reads were converted to fastq format using mkfastq from Cell Ranger 2.1.0 (10× Genomics, San Francisco, CA, USA). Reads were then aligned to the human reference (hg38) and mouse reference genomes (mm10). Alignment was performed using the count command from Cell Ranger 2.1.0 (10× Genomics).

R 4.0.5 (R Foundation for Statistical Computing, Vienna, Austria; https://www.r-project.org/) and Seurat 4.0.1 were used for downstream analyses.^(^ ^17^ ^)^ Cells with <1000 expressed genes or with more than a 10% fraction of expressed mitochondrial genes in the total expressed genes were excluded. The cell‐cycle score in each cell was calculated based on cell‐cycle genes in the 2019 updated version. Cell‐cycle scores and the proportional rate of mitochondrial genes among the total expressed genes were regressed out using scTransform.^(^ ^18^ ^)^

For the interspecies analysis, gene symbols were transformed to the corresponding homolog gene names using a gene conversion table called geneTrans.^(^ ^19^ ^)^ Human and humanized murine or murinized human and murine data sets were merged, then the merged data sets were divided according to their origin and were regressed out of cell‐cycle scores and expressed mitochondrial gene's proportion. After that, the two divided data sets were integrated based on 5000 common anchoring genes selected using the “SelectIntegrationFeatures” command.

After “RunPCA,” dimension in “FindNeighbors” was determined manually referring to the shape of elbow plot and tidy distribution of cells into clusters illustrated by clustree,^(^ ^20^ ^)^ which is useful for determining a reasonable resolution in “FindClusters” with a modified Louvain algorithm.^(^ ^21^ ^)^ The criteria used to choose a resolution were as follows: first, the resolution is 1, or near 1, which is the standard resolution with the algorithm. Second, diagonal arrows at a resolution on clustree are absent or few. Third, cells are distributed properly on Uniform Manifold Approximation and Projection (UMAP).^(^ ^22^ ^)^ Finally, the annotation in each cluster should be meaningful.

Dot plots, feature plots, violin plots, and heat maps were drawn using Seurat. A trajectory map was generated with Monocle 3 using UMAP made by Seurat as a mold. To find differentially expressed genes (DEGs) in each cluster, “FindAllMarkers” was used with a logfc. threshold 0.30 and min.pct 0.20. The top 25 DEGs in each cluster were selected with an adjusted *p* value. In order to compare Gene Ontology (GO) term activity among clusters, clusterProfiler was used.^(^ ^23^ ^)^ By taking intracellular transcriptional regulations and targets into consideration, relationships between extracellular ligands and receptors on preosteoclasts and osteoclasts could be depicted using NicheNet.^(^ ^24^ ^)^ To extract the marker molecules in each cluster, the results of “FindAllMarkers” were narrowed down by cluster of differentiation (CD) molecules, chemokines, and their receptors. They were illustrated using ggplot2.^(^ ^25^ ^)^

### Chromatin immunoprecipitation sequencing

Cells were prepared as previously described.^(^ ^3^ ^)^ For chromatin immunoprecipitation (ChIP), anti‐H3K4me3, anti‐H3K27me3, and anti‐H3K27ac antibodies (Diagenode, Seraing, Belgium) and anti‐Nfatc1 antibody (Santa Cruz Biotechnology, Dallas, TX, USA) were used. Briefly, cells were fixed in 1% formaldehyde and neutralized with glycine. After sonicating the cells, the samples were incubated with a mixture of protein A and protein G beads that were preincubated with 4 to 10 μg of the respective antibodies. After washing the samples several times, reverse cross‐linking was performed. DNA was purified using a PCR purification kit (Qiagen, Germantown, MD, USA). A DNA library was generated according to the manufacturer's instructions included with the Illumina protocol. Single‐end reads of 100 bp were generated using an Illumina HiSeq 2500 system. Alignment to the GRCh37 reference genome was performed with bwa mem v0.7.14‐r1136. MACS version 2.1.1.20160309 was used to call peaks for each sample, using both input and immunoglobulin G (IgG) alignments as controls. In preparation for a motif enrichment search, two different region files were generated from the peak files as output by MACS. First, a file that combined all three peak files, then a file with only the flanking regions (200 bp in either direction) of each peak region in the combined file (vicinity analysis). Motif enrichment analysis was then performed using HOMER software version 4.9.1. For region of interest (ROI)‐based ChIP‐seq peak and RNA‐seq overlap analysis, ROIs were defined as regions 225‐kb upstream and downstream of differentially expressed genes. Within each ROI, the most significantly enriched peak from ChIP‐seq analysis was determined.

### Quantitative real‐time PCR

Cells were transferred to TRIzol (Invitrogen, Carlsbad, CA, USA), and RNA was extracted and reverse transcribed according to the manufacturer's instructions using a QuantiTect Reverse Transcription kit (QIAGEN, Valencia, CA, USA). PCR was conducted using an ABI Prism 7000 Sequence Detection System (Applied Biosystems, Foster City, CA, USA) with QuantiTect SYBR Green (QIAGEN, Valencia, CA, USA). All reactions were performed in triplicate. Gene expression was measured as arbitrary units relative to the expression of actin. Primer sequences were as follows: human Cathepsin K_forward, 5′‐AGAAGACCCACAGGAAGCAA‐3′, human Cathepsin K_reverse, 5′‐GCCTCAAGGTTATGGATGGA‐3; human NFATc1_forward, 5′‐GTCCTGTCTGGCCACAAC‐3′, human NFATc1_reverse, 5′‐GGTCAGTTTTCGCTTCCATC‐3; murine Cathepsin K_forward, 5′‐ACGGAGGCATTGACTCTGAAGATG‐3, murine Cathepsin K_reverse; 5′‐GGAAGCACCAACGAGAGGAGAAAT‐3; murine Nfatc1_forward, 5′‐ GGGTCAGTGTGACCGAAGAT‐3, murine Nfatc1_reverse; 5′‐GGAAGTCAGAAGTGGGTGGA‐3; murine Rab38_forward, 5′‐CCAAGGGAAGGATGTGCTTA‐3, murine Rab38_reverse; 5′‐GTGAGATGGGGCTTCACAAT−3; murine Rab12_forward, 5′‐GAAGTGCCAAGGGGATCATA‐3, murine Rab12_reverse; 5′‐TCCTTGCTGCCTTGAGATTT‐3; murine Rab18_forward, 5′‐TGCACGCAAGCATTCTATGT‐3, murine Rab18_reverse; 5′‐GGCTCTCTTCCCTGTGTGAC‐3; murine Rab34_forward, 5′‐CCAGTGCCTGAAGAAAGAGG‐3, murine Rab12_reverse; 5′‐GGGACACCCAAGACTTCAAA‐3.

### Immunocytochemistry assay

Murine osteoclasts were generated by culturing murine bone marrow‐derived macrophages (BMMs), as described.^(6)^ Cells were immunostained with the primary antibody, anti‐Rab38 (Proteintech, Rosemont, IL, USA), followed by the secondary antibody, Alexa Fluor 488 (Invitrogen, Thermo Fisher Scientific, Waltham, MA, USA), then counterstained with 4′,6‐diamidino‐2‐phenylindole (DAPI). Cells were observed under fluorescence microscopy (BZ‐8100; Keyence, Osaka, Japan). Cells were incubated for 30 minutes with rhodamine‐conjugated phalloidin solution (Molecular Probes, Eugene, OR, USA) for staining F‐actin. For measurement of actin ring formation rate, intact/total circumferences were measured by using ImageJ software (NIH, Bethesda, MD, USA; https://imagej.nih.gov/ij/).

### In vitro gene silencing by short hairpin RNA

In vitro gene silencing was planned before the stimulation of RANKL. Short hairpin RNA (shRNA) expression vectors were constructed using piGENEmU6 vector (iGENE Therapeutics, Tokyo, Japan), as described,^(^ ^26^ ^)^ and the U6 promoter and inserts were cloned into retroviral plasmid vectors. Retroviruses carrying specific genes were prepared by transfecting human kidney cell line packaging cells with retrovirus vectors and collecting the supernatant after 2 days. For retroviral infection, after the first 2 days of culture, BMMs were incubated with retrovirus in the presence of 10 ng/mL M‐CSF and 4 ng/mL polybrene (Sigma‐Aldrich, St. Louis, MO, USA) for 6 hours and cultured overnight in the presence of 10 ng/mL M‐CSF. To select the transduced BMMs, cells were detached with trypsin/EDTA (Sigma‐Aldrich) and cultured with 10 ng/mL M‐CSF and 2 μg/mL puromycin (Sigma‐Aldrich) for 2 days. After cell selection with puromycin, the surviving cells were cultured with 10 ng/mL M‐CSF and 100 ng/mL RANKL. The sequence of the construct for shRNA was as follows: Rab38 shRNA‐1F: gtttAaACACCcCACAAaGAGCACCcGTAgtgtgctgtccTACAGGTGCTCCTTGTGAGGTGTCTttttt, R: atgcaaaaaAGACACCTC.

ACAAGGAGCACCTGTAggacagcacacTACAaaTGCcCCTcGTaAGaTGcCT; Rab38 shRNA‐2 F: gtttCACCcCACAAcaAcCACCcGTACAAgtgtgctgtccTTGTACAGGTGCTCCTTGTGAGGTttttt, R: atgcaaaaaCACCTCACAAGGAGCACCTGTACAAggacagcacacTcGcACAaGTaCTCCcTGcGAaGT.

### In vitro gene silencing by Cas9/guide RNA

Murine bone marrow‐derived macrophages were transfected by Cas9 lentiviral particles (Invitrogen, Carlsbad, CA, USA; A32064) at 70% to 80% confluency in the M‐CSF‐containing medium according to the instruction. Briefly, the cells were transduced with Cas9 lentivirus and guide RNA of GFP control (Invitrogen; A32060) or Rab38 (Invitrogen; CRISPR95930_SGM), at a multiplicity of infection (MOI) of 2, in the presence of 5 μg/mL polybrene (Merck Millipore, Burlington, MA, USA). After 10 hours of incubation, the medium was changed to M‐CSF and RANKL‐containing medium for generating osteoclastogenesis. The target sequence of the guide RNA (gRNA) for murine Rab38 is GGTAGTGCGAGGAGAAGTTT (Invitrogen, CRISPR95930_SGM).

### Statistical analysis

Statistical analyses were performed using Student's *t* test, the Mann‐Whitney test, and one‐way or two‐way ANOVA followed by Tukey's multiple‐comparison post‐test. The star marks *, **, and *** are indicated as *p* < 0.05, *p* < 0.01, or *p* < 0.001, respectively. Prism Software Version 8 (GraphPad Software, Inc., La Jolla, CA, USA) was used for the generation of graphs and the statistical analyses.

## Results

**Fig. 1:**
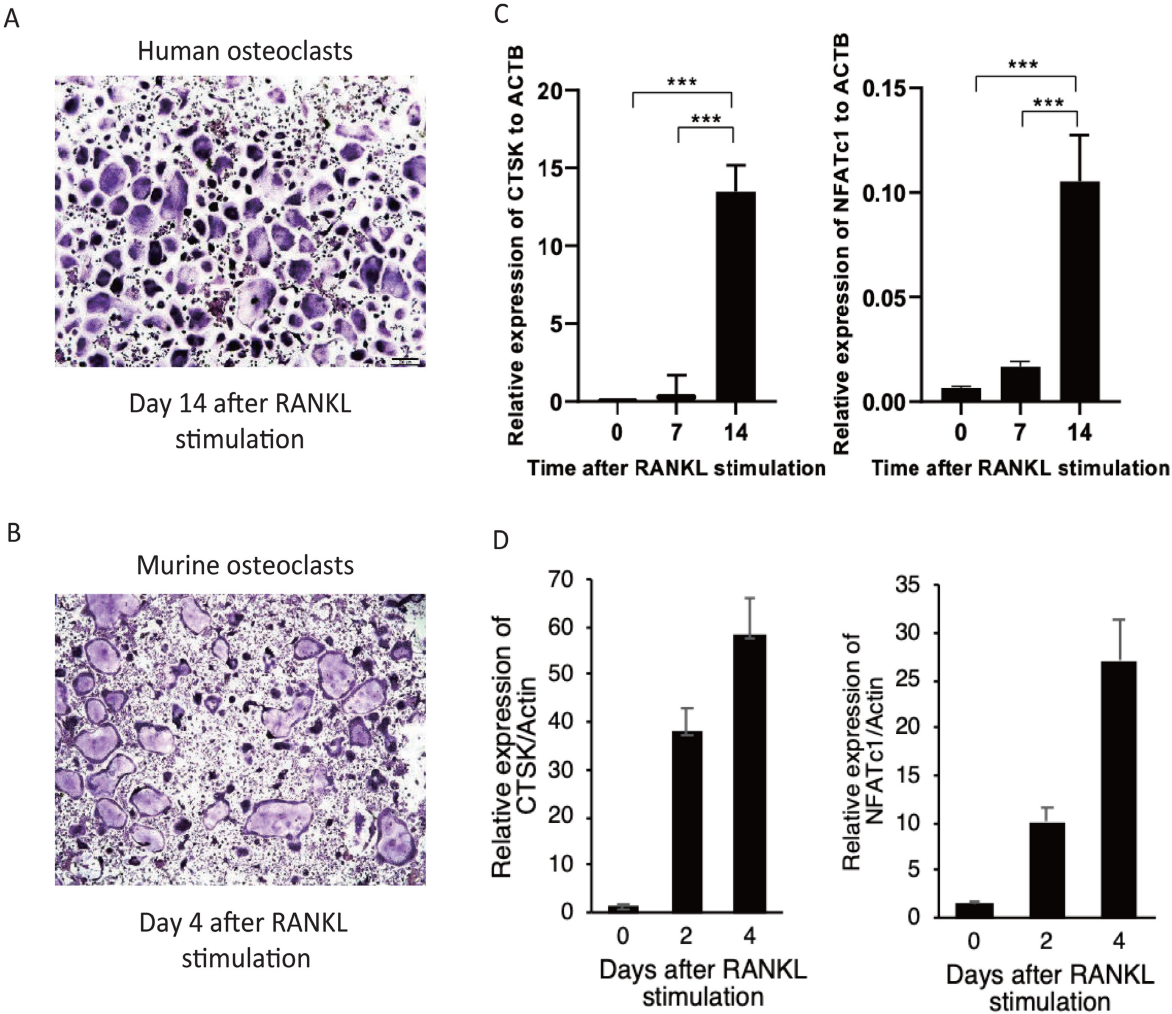
In vitro human and murine osteoclast differentiation following stimulation with recombinant M‐CSF and RANKL. TRAP staining in mature human (*A*) and murine (*B*) osteoclasts (scale bar = 200 μm). The expression of differentiation factors in human (*C*) and murine (*D*) osteoclasts. The values were measured in triplicate and compared against day 0 (***p < 0.001). M‐CSF = macrophage colony‐stimulating factor; RANKL = receptor activator of nuclear factor‐kappa‐Β ligand; TRAP = tartrate‐resistant acid phosphatase.

As shown in Fig. 1*A*,*B*, TRAP‐positive, mature osteoclasts were induced from human PBMCs and murine bone marrow cells in response to RANKL and M‐CSF treatment. Longer incubation periods were required for human osteoclastogenesis. The induction of osteoclast‐specific genes, such as *CTSK* and *NFATc1*, was confirmed by quantitative PCR analysis (Fig. 1*C*,*D*). As shown in Fig. 1*A*,*C*, not all cells differentiated into mature osteoclasts, suggesting that the cultures contained heterogeneous OLCs during the differentiation.

**Fig. 2:**
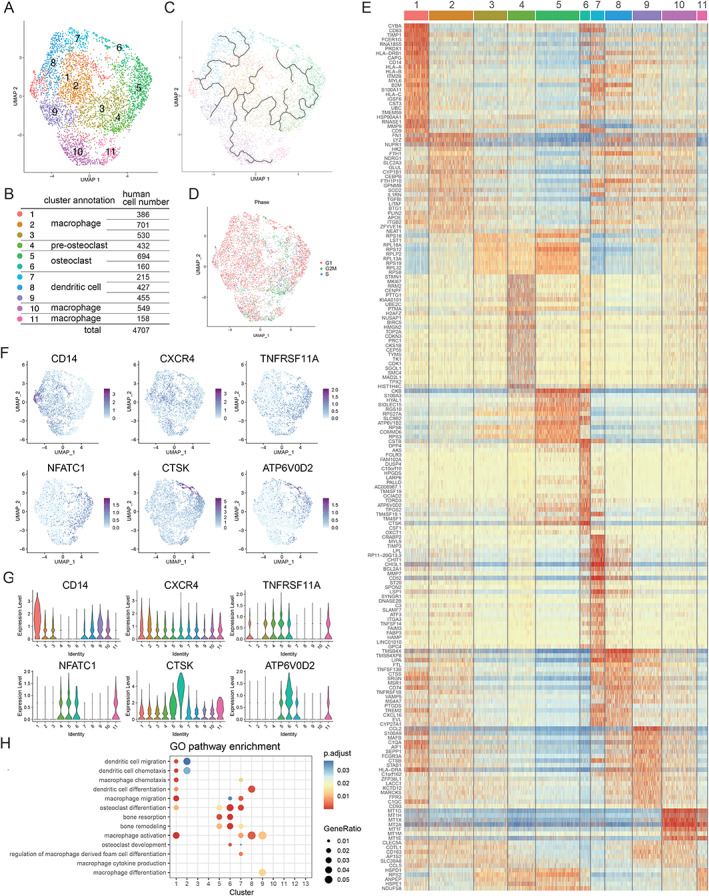
Transcriptional profiling of human osteoclastogenesis by scRNA‐seq. In vitro cultured human mature osteoclasts generated by stimulation with recombinant M‐CSF and RANKL were analyzed by scRNA‐seq. (*A*) Feature plot (UMAP) demonstrating a total of 11 clusters during human osteoclastogenesis (*n* = 4707 cells). (*B*) Cluster annotation and cell number of each cluster in the human OLC data set. (*C*) Cell‐based trajectory analysis in human osteoclastogenesis using Monocle 3 in UMAP. (*D*) Phase analysis of cell‐cycle status (red, G1; green, G2/M; blue, S phase). (*E*) Heat map of the top 25 differentially expressed genes in each OLC cluster. Osteoclastic markers *CTSK* and *ATP6V0D2* are highly expressed in cluster 6. (*F*,*G*) Feature plot (*F*) and violin plot (*G*) of marker genes. *CD14* and *CXCR4* are progenitor markers. *TNFRSF11A*, *NFATc1*, *CTSK*, and *ATP6V0D2* are osteoclast‐associated genes. (*H*) Dot plot of GO terms enriched in each cluster related to “dendritic cell,” “macrophage,” “osteoclast,” and “bone.” GO = gene ontology; M‐CSF = macrophage colony‐stimulating factor; RANKL = receptor activator of nuclear factor‐kappa‐Β ligand.

**Fig. 3:**
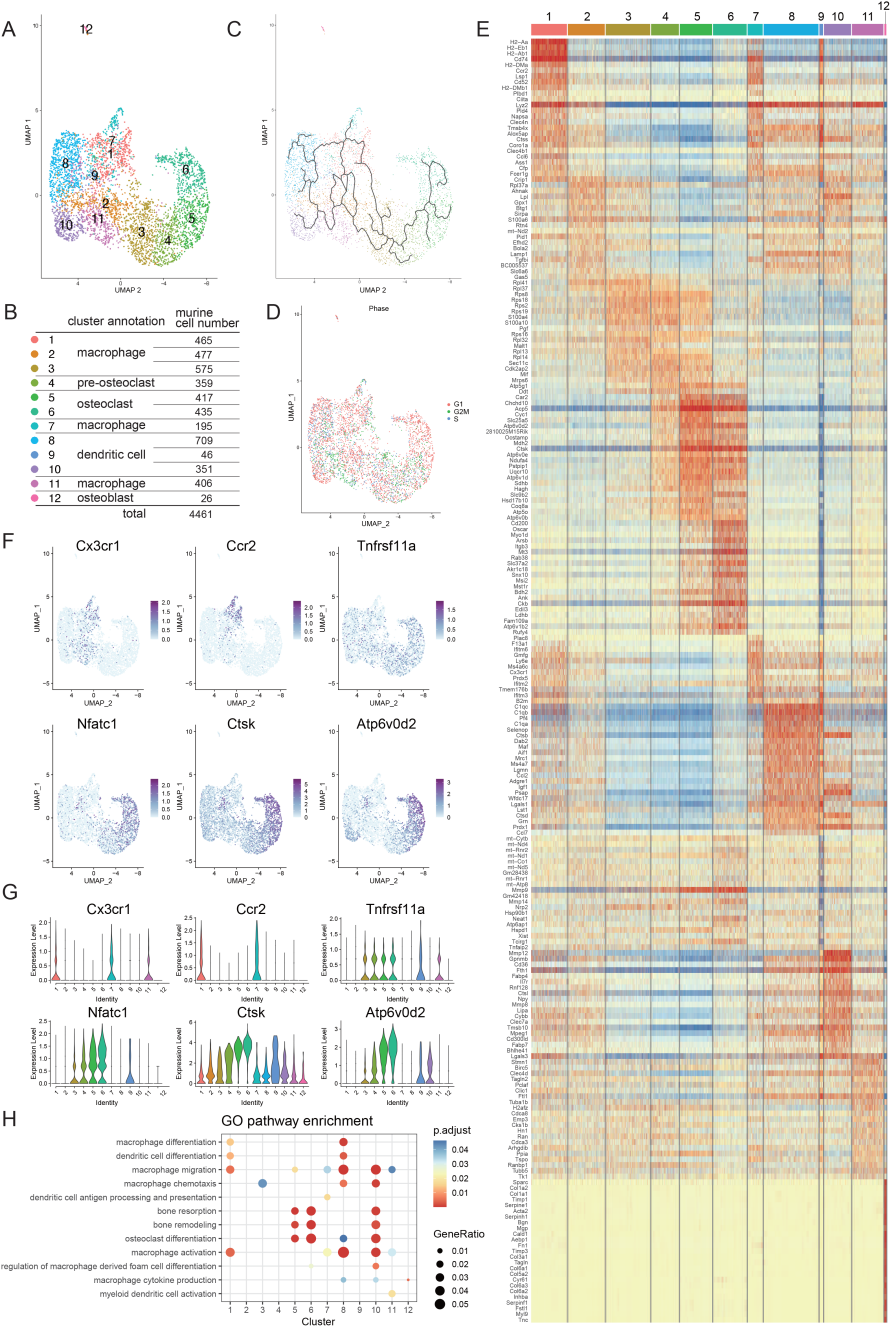
Transcriptional profiling of murine osteoclastogenesis by scRNA‐seq. In vitro cultured murine mature osteoclasts generated by stimulation with recombinant M‐CSF and RANKL were analyzed by scRNA‐seq. (*A*) Cell‐based trajectory analysis in murine osteoclastogenesis. (*B*) Cluster annotation and cell number of each cluster in the murine OLC data set. (*C*) Pseudo‐time analysis shows a cell‐based trajectory in murine osteoclastogenesis. (*D*) Phase analysis of cell‐cycle status (red, G1; green, G2/M; blue, S phase). (*E*) Heat map of the top 25 differentially expressed genes in each OLC cluster. Osteoclastic markers *Acp5*, *Ocstamp*, *Ctsk*, and *Atp6v0d2* are highly expressed in cluster 6. (*F*,*G*) Feature plot (*F*) and violin plot (*G*) of marker genes. *Cx3cr1* and *Ccr2* are progenitor markers. *Tnfrsf11a*, *Nfatc1*, *Ctsk*, and *Atp6v0d2* are osteoclast‐associated genes. (*H*) Dot plot of gene ontology terms enriched in each cluster related to “dendritic cell,” “macrophage,” “osteoclast,” and “bone.” M‐CSF = macrophage colony‐stimulating factor; RANKL = receptor activator of nuclear factor‐kappa‐Β ligand.

To better understand the heterogeneity of OLCs and detailed trajectories of osteoclast differentiation, we investigated human and murine osteoclast cultures at the single cell level using scRNA‐seq analysis. We first analyzed in vitro differentiated human and murine mature osteoclast cultures using 10× Genomics tools (Fig. S1 *A*). Subsequent bioinformatics analyses were conducted using Seurat and Monocle to perform UMAP to analyze the clustering and trajectory of OLCs (Figs. 2*A*–*E*, and 3*A*‐*E*, Figs. 2*A*–*C* and 3*A*–*C*). Based on the feature plot (UMAP) analysis (Figs. 2*A* and 3*A*), in total we identified 11 clusters of human OLCs and 12 clusters of murine OLCs (Figs. 2*B* and 3*B*). As shown in Figs. 2*C* and 3*C*, mature osteoclasts (clusters 5 and 6) were differentiated from the monocyte–macrophage lineage cells (cluster 1), but not from cells in the mitotic G2/M phase (cluster 11) (Figs. 2*D* and 3*D*). Heat maps (Figs. 2*E* and 3*E*) showed that osteoclast‐specific genes, such as *ATP6V0D2*, *CTSK*, *ACP5*, and *OCSTAMP*, are highly expressed at cluster 6, whereas known osteoclast‐related genes, such as *CD14*, *CXCR4*, *Cx3cr1*, *Ccr2*, *TNFRSF11A*, *NFATc1*, *CTSK*, and *ATP6V0D2*, are expressed at different stages of OLCs (Figs. 2*F*,*G* and 3*F*,*G*). *CD14* and *CXCR4* are expressed in clusters 1 and 2 of human OLCs. *Cx3cr1* and *Ccr2* are expressed in clusters 1 and 7 of murine OLCs. *TNFRSF11A*, *NFATc1*, *CTSK*, and *ATP6V0D2* are expressed in clusters 4 to 6. Based on the expression of osteoclast‐specific genes, clusters 5 and 6 were defined as mature osteoclasts in both human and murine scRNA‐seq datasets. GO analyses (Figs. 2*H* and 3*H*, Figs. S2*F* and S3*F*) also showed that clusters 5 and 6 were associated with GO terms related to osteoclasts such as “osteoclast differentiation,” “bone resorption,” and “bone remodeling.” The expression of osteoclast‐specific genes such as *CTSK* and *ATP6V0D2* in cluster 6 was higher than that in cluster 5, indicating that cluster 6 represents a fully differentiated osteoclast cluster. It should be noted that the detailed analysis of the heat maps showed different gene profiles between corresponding human and murine clusters.

**Fig. 4:**
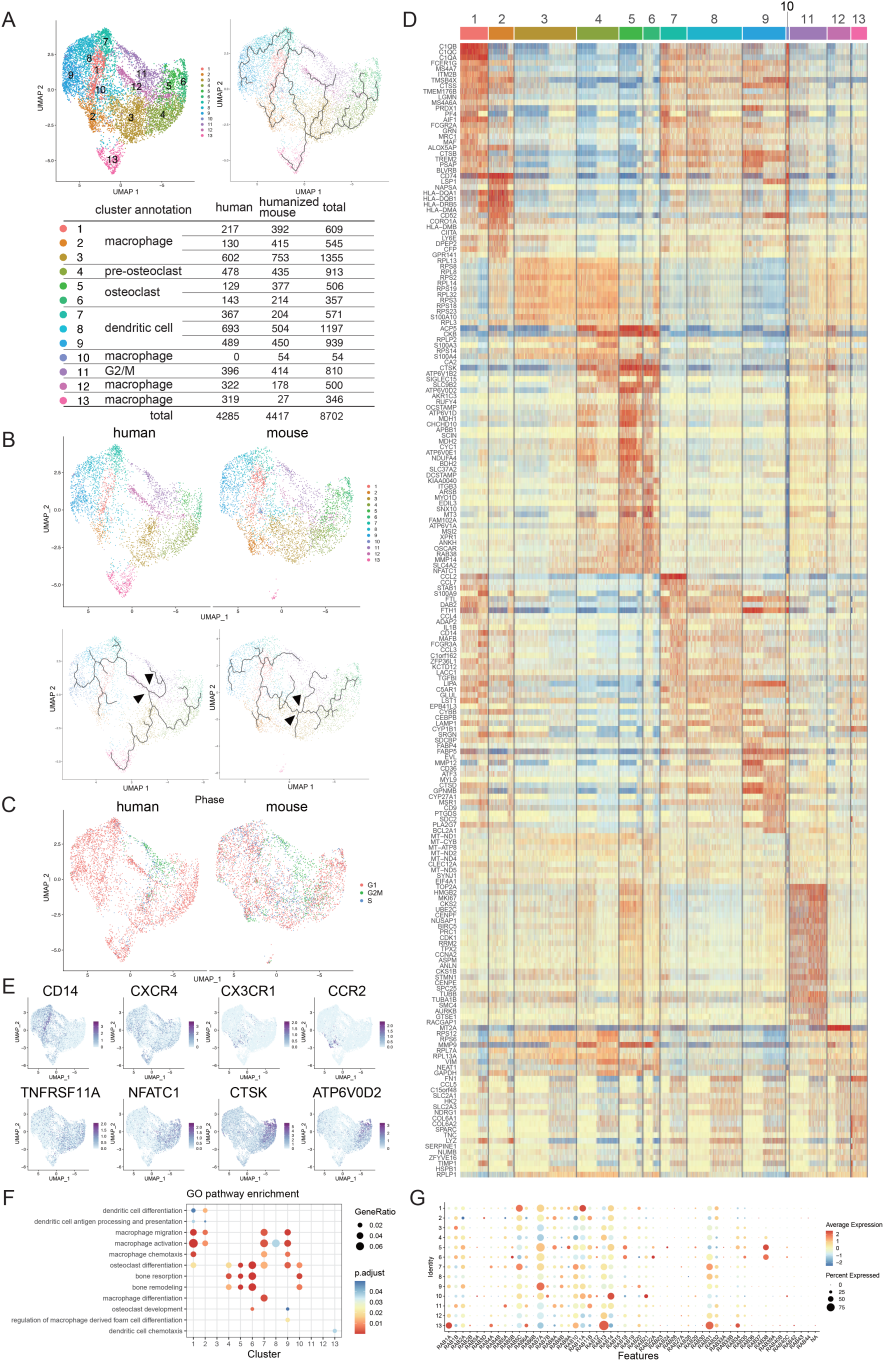
Analysis of integrated data from human and humanized murine osteoclast differentiation. Human and humanized mouse scRNA‐seq data were integrated. (*A*) Feature plot (UMAP) demonstrating a total of 13 clusters during osteoclastogenesis (human, *n* = 4285; humanized mouse, *n* = 4417; total, *n* = 8702 cells). Cell‐based trajectory analysis of the integrated data set. Cluster annotation and cell number of each cluster in the human and humanized murine OLC data set. (*B*) Feature plots and cell‐based trajectory in the reconstructed human and murine OLC data set. Arrows indicate the branch into OLC. (*C*) Phase analysis of cell‐cycle status in the reconstructed human and murine OLC data set (red, G1; green, G2/M; blue, S phase). (*D*) Heat map of the top 25 differentially expressed genes in each OLC cluster. Osteoclastic markers *ACP5*, *CTSK*, *ATP6V0D2*, *OCSTAMP*, *DCSTAMP*, *OSCAR*, and *NFATC1* are highly expressed in clusters 5 and 6. (*E*) Feature plot of marker genes. *CD14* and *CXCR4* are progenitor markers. *TNFRSF11A*, *NFATc1*, *CTSK*, and *ATP6V0D2* are osteoclast‐associated genes. (*F*) Dot plot of GO terms enriched in each cluster related to “dendritic cell,” “macrophage,” “osteoclast,” and “bone.” (*G*) Expression of detectable RAB family genes in the integrated data set. Note that RAB38 is predominantly expressed in osteoclastic clusters 5 and 6.

**Fig. 5:**
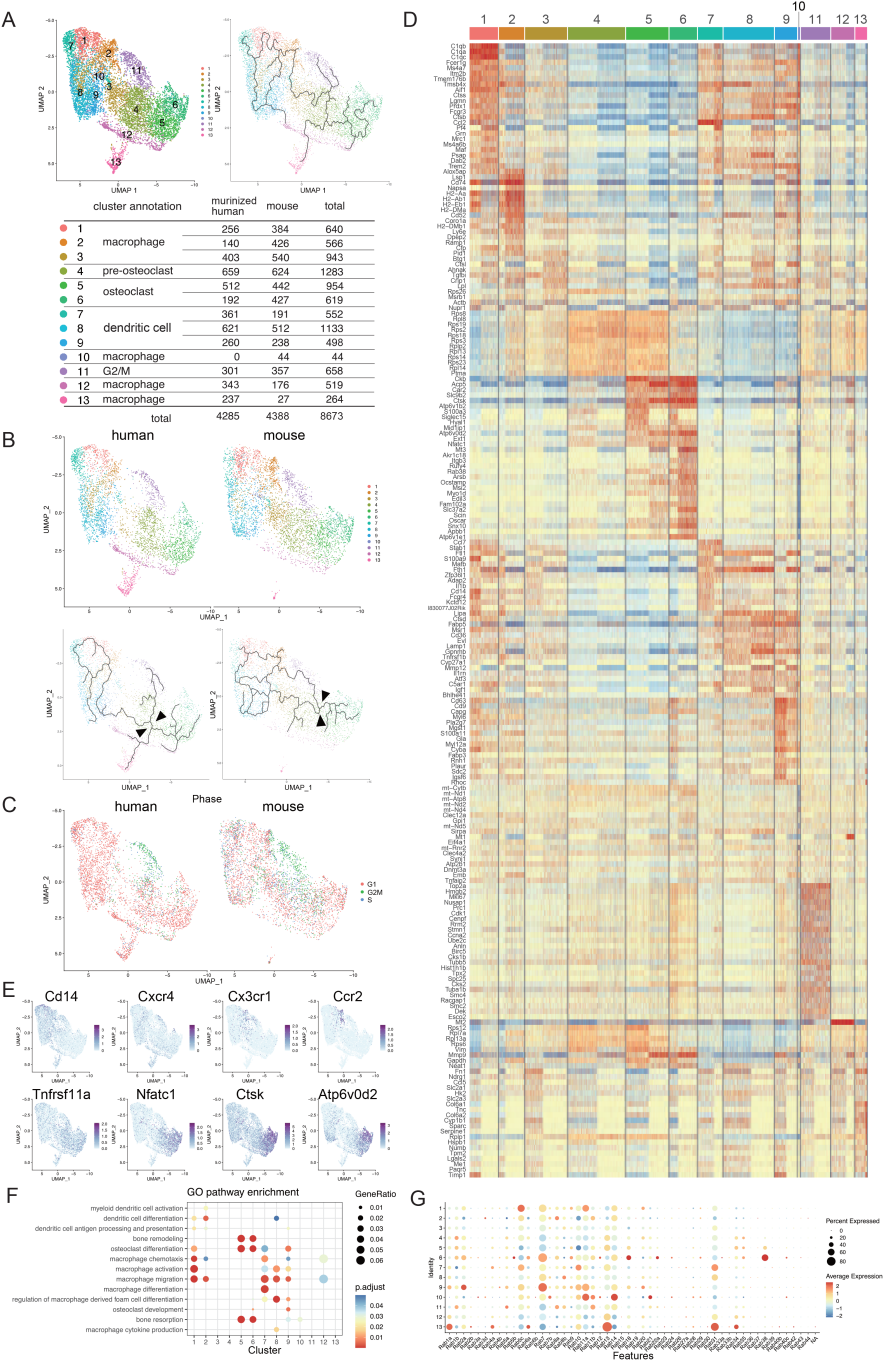
Analysis of integrated data from murinized human and murine osteoclast differentiation. Murinized human and murine scRNA‐seq data were integrated. (*A*) Feature plot (UMAP) demonstrating a total of 13 clusters during osteoclastogenesis (murinized human, *n* = 4285; mouse, *n* = 4388; total, *n* = 8673 cells). Cell‐based trajectory analysis in the integrated data set. Clusters and annotation in the murinized human and murine OLC data set. (*B*) Feature plots and cell‐based trajectory in the reconstructed human and murine OLC data set. Arrows indicate the branch into OLC. (*C*) Phase analysis of cell‐cycle status in the reconstructed human and mouse OLC data set (red, G1; green, G2/M; blue, S phase). (*D*) Heat map analysis of the top 25 differentially expressed genes in each OLC cluster. Osteoclastic markers *Acp5*, *Ctsk*, *Atpv0d2*, *Nfatc1*, *Ocstamp*, and *Oscar* are highly expressed in clusters 5 and 6. (*E*) Feature plot of marker genes. *Cd14*, *Cxcr4*, *Cx3cr1*, and *Ccr2* are progenitor markers. *Tnfrsf11A*, *Nfatc1*, *Ctsk*, and *Atp6v0d2* are osteoclast‐associated genes. (*F*) Dot plot of GO terms enriched in each cluster related to “dendritic cell,” “macrophage,” “osteoclast,” and “bone.” (*G*) Expression of detectable Rab family genes in the integrated data set. Note that Rab38 is predominantly expressed in osteoclastic clusters 5 and 6.

To further analyze the similarities and differences between human and murine osteoclastogenesis, we integrated human and murine scRNA‐seq data into a single data set (Figs. 4 and 5, Figs. S1 *B*, S4, and S5). Based on the feature plot (UMAP) analysis (Figs. 4*A* and 5*A*), we distinguished a total of 13 clusters for the integrated human and murine OLCs. As shown in Figs. 4*B* and 5*B*, mature osteoclasts (cluster 6) differentiated from the monocyte/macrophage lineage cells (cluster 1), as shown in both human and murine OLC analyses. However, trajectory analyses revealed differences between human and murine OLCs. The trajectories branched at early stages in human OLCs but at later stages in murine OLCs, although both trajectories ultimately proceeded in the same direction toward mature osteoclasts. Although the number of cells in cluster 4 was similar between humans and mice (Figs. 4*A* and 5*A*), most of the human OLCs in cluster 4 were in G1 phase, whereas most of those in mice were in G1/S phase. Furthermore, the OLCs in the mitotic G2/M phase (cluster 11) did not differentiate into mature osteoclasts (Figs. 4*A*–*C* and 5*A*–*C*). Heat maps of the top 20 expressed genes in each cluster showed high levels of expression of osteoclast‐specific markers, such as *ACP5*, *CTSK*, *ATP6V0D2*, *OCSTAMP*, *DCSTAMP*, *OSCAR*, and *NFATc1*, in clusters 5 and 6 (Figs. 4*D* and 5*D*).

To analyze the characteristics of the mature osteoclast clusters, we performed GO Biological Process analysis (Figs. 4*F* and 5*F*, Figs. S3*F* and S4*F*), and found that clusters 5 and 6 were associated with osteoclast‐related terms such as “osteoclast differentiation,” “bone resorption,” and “bone remodeling.”

The ligand–receptor analysis exhibited specific ligand–receptor interactions involved in both human and murine osteoclastogenesis (Fig. S6). For example, colony‐stimulating factor 1 (CSF1)/CSF1 receptor (CSF1R), TGF‐β1/TGF receptor (TGFR), C‐C motif chemokine ligand 2 (CCL2)/C‐C motif chemokine receptor 1 (CCR1), and CCL3/CCR1 were extracted as high‐affinity interactions. The additional analyses of major markers revealed that some known osteoclast‐related molecules were upregulated in cluster 6 (Fig. S7). For example, well‐known osteoclast regulators, such as *ITGB3*, *CD68*, *SEMA4D*, *CCR1*, and *CD200*, were upregulated in each data set.^(^ ^27^, ^28^, ^29^, ^30^, ^31^ ^)^

The Rab GTPase family belongs to the Ras superfamily of small GTPases. The Rab family contains at least 70 members that have emerged as central regulators of intracellular trafficking signals. They possess multiple binding domains for regulatory molecules and downstream effectors; they also act as scaffold proteins. Previous studies have demonstrated that several Rab family members are expressed in osteoclasts.^(^ ^32^, ^33^ ^)^ To identify Rab family members that play a critical role in osteoclast differentiation, we comprehensively analyzed gene expression profiles of Rab family proteins in the integrated data set of OLCs. As shown in the dot plots and feature plots (Figs. 4*G* and 5*G*, Figs. S4 and S5*E*), *RAB38* was predominantly expressed not only in the separate human and murine data (Figs. S2*D*,*E* and S3*D*,*E*) but also in the integrated data (clusters 5 and 6).

**Fig. 6:**
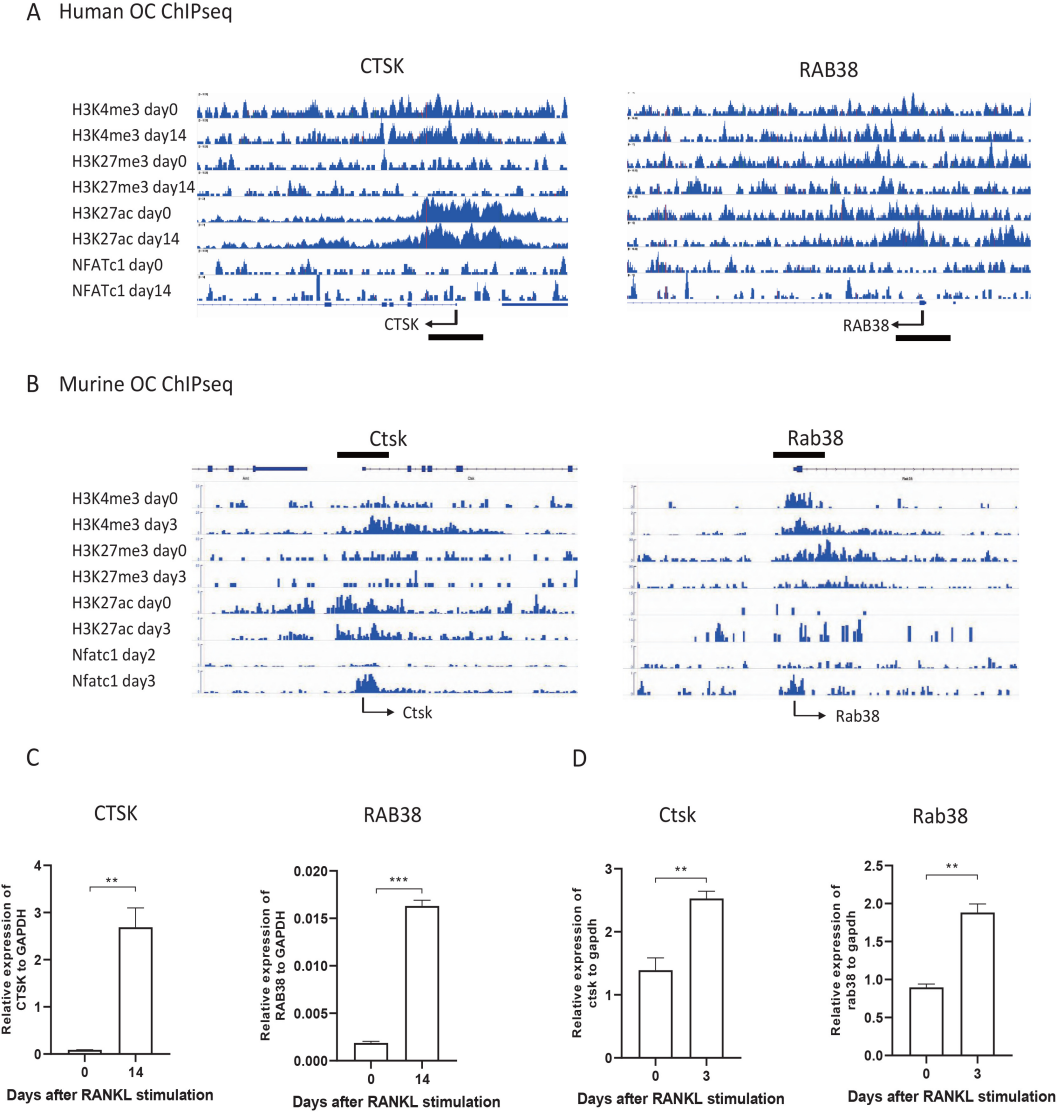
ChIP‐seq profiling of human and murine osteoclast precursors and mature osteoclasts. Data represent the ChIP‐seq results for the active chromatin marker H3K4me3 (histone H3 lysine 4 trimethylation), the repressive chromatin marker H3K27me3 (histone H3 lysine 27 trimethylation), the active enhancer marker H3K27ac (histone H3 lysine 27 acetylation), and the osteoclast‐specific marker NfatC1. (*A*) Histone methylation and acetylation status and the binding of NFATc1 at the *Ctsk* and *Rab38* genes, as analyzed by ChIP‐seq from day 0 to day 14 following RANKL stimulation in human osteoclast precursors and mature osteoclasts. The histone modification patterns changed from H3K4me3/H3K27me3 bivalent to H3K4me3 monovalent at both *Ctsk* and *Rab38* following RANKL stimulation. The histone acetylation H3K27ac and the binding of NFATc1 increased at both the *Ctsk* and *Rab38* gene regions. Bold bars indicate +/− 1 Kb from TSS. (*B*) Histone methylation and acetylation status and the binding of NFATc1 at the *Ctsk* and *Rab38* genes were analyzed by ChIP‐seq at day 0 or day 2 and day 3 following RANKL stimulation in murine osteoclast precursors and mature osteoclasts. The level of H3K4me3 increased at the *Ctsk* gene. The histone modification patterns changed from H3K4me3/H3K27me3 bivalent to the H3K4me3 monovalent at the *Rab38* gene following RANKL stimulation. The histone acetylation H3K27ac increased at the *Rab38* gene, and the binding of NFATc1 increased at both the *Ctsk* and *Rab38* gene regions. Bold bars indicate +/− 1Kb from TSS. (*C*,*D*) Gene expression was analyzed by RNA‐seq in human and murine osteoclast precursors and mature osteoclasts. *Ctsk* and *Rab38* gene expression was analyzed by RNA‐seq at day 0 and day 14 following RANKL stimulation to induce human osteoclast differentiation. Gene expression of *Ctsk* and *Rab38* was analyzed by RNA‐seq at day 0 and day 3 following RANKL stimulation during murine osteoclast differentiation. ChIP‐seq = chromatin immunoprecipitation‐sequencing; RANKL = receptor activator of nuclear factor‐kappa‐Β ligand; TSS = transcription start site.

We performed ChIP‐seq to profile the histone modifications associated with the *RAB38* gene during human and murine osteoclastogenesis (Fig. 6*A*,*B*). The ChIP‐seq analysis of lysine 4 trimethylation of histone H3 (H3K4me3), H3K27me3, lysine 27 acetylation of H3 (H3K27ac), and NFATc1 binding demonstrated that histone modification patterns changed from H3K4me3/H3K27me3 bivalent to H3K4me3 monovalent patterns at the *cis*‐regulatory regions of both *CTSK* and *RAB38* genes in response to RANKL stimulation, indicating the transcriptional activation of these genes. In addition, H3K27ac modification and the binding of NFATc1 were also increased at the promoter/enhancer regions of *CTSK* and *RAB38* genes. Conventional RNA‐seq analyses revealed that *RAB38* gene expression was increased by RANKL stimulation in both human and murine OLCs (Fig. 6*C,D*). These results suggest that *RAB38* plays a critical role during human and murine osteoclast differentiation.

**Fig. 7:**
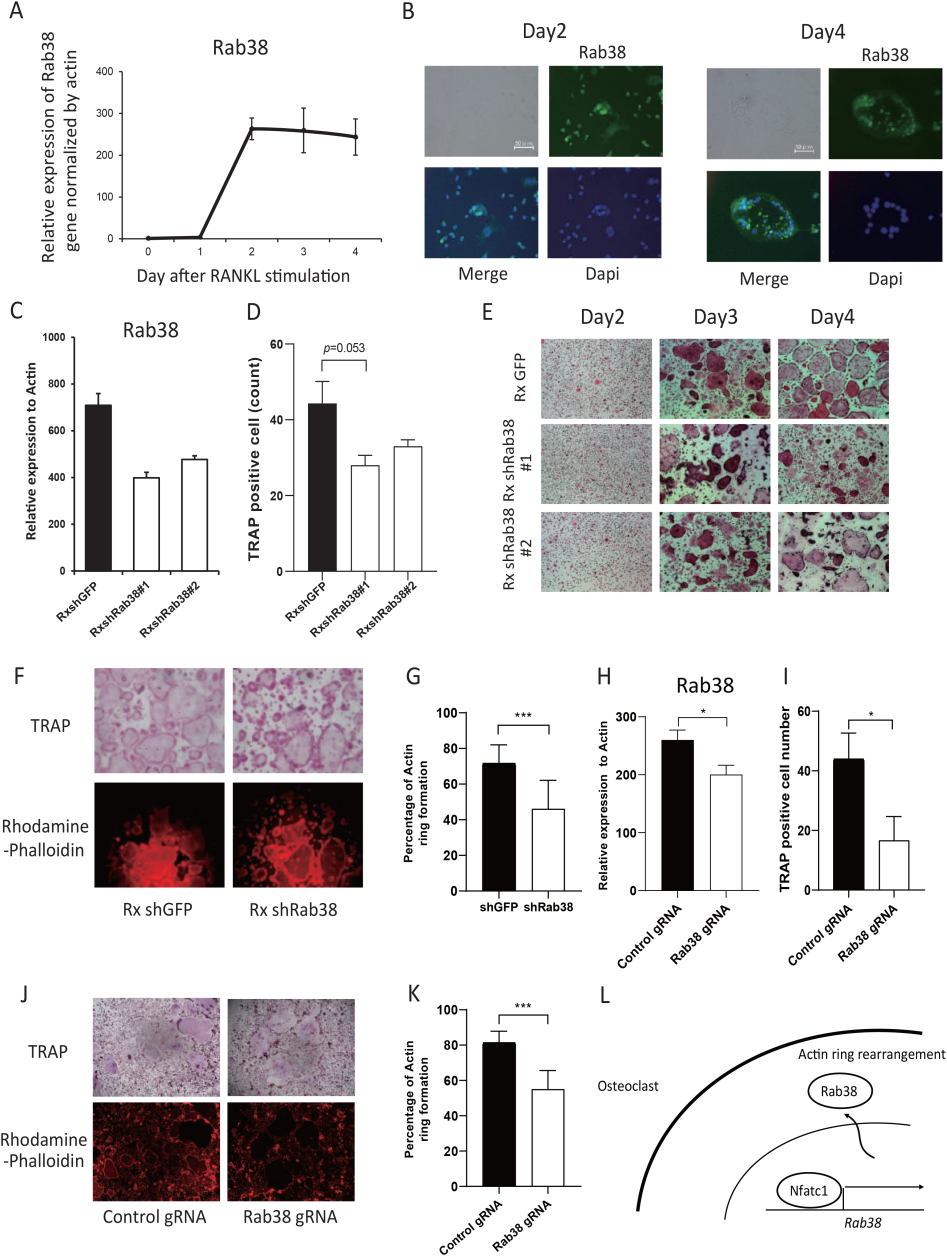
Effect of *Rab38* knockdown by retroviral shRNA on murine osteoclast differentiation. (*A*) Expression of *Rab38* genes in mouse osteoclast differentiation measured by quantitative real‐time PCR. (*B*) Expression of Rab38 protein in mature osteoclasts (green; Rab38, blue; nucleus; scale bar indicates 50 μm). (*C*,*H*) Expression of *Rab38* gene under conditions of *Rab38* knockdown by retroviral shRNA (*C*) or by lentiviral Cas9/gRNA (*H*) and the controls. (*D*,*I*) Counts of TRAP‐positive cells (≥3 nuclei). (*E*,*F*,*J*) TRAP staining (*E*,*F*,*J*) and cytoskeletal staining by rhodamine‐phalloidin (*F*,*J*) of osteoclasts under the respective conditions of *Rab38* knockdown. (*G*,*K*) Actin ring formation rate. Ten cells in each condition were measured. (*L*) Schematic representation of the regulation of osteoclastogenesis by the Nfatc1–Rab38 axis. The values were measured in triplicate and compared against control (*p < 0.05, ***p < 0.001).

The sequential expression analysis using quantitative PCR of *Rab38* during murine osteoclastogenesis showed that its expression increased 2 days following RANKL stimulation (Fig. 7*A*). In the immunohistochemistry analysis shown in Fig. 7*B*, Rab38 was expressed in the nuclei on day 2 and then translocated to the cytoplasm. Furthermore, the role of Rab38 was examined by suppressing *Rab38* gene expression using a retrovirus‐mediated shRNA targeting Rab38 or using a lentivirus‐mediated Cas9/guided RNA of Rab38 in murine OCPs (Fig. 7*C*–*K*). Not only was osteoclast differentiation inhibited but actin ring formation in mature osteoclasts was also markedly impaired when Rab38 was suppressed by shRNA or Cas9/gRNA, indicating an essential role for Rab38 in both differentiation and activation of osteoclasts (Fig. 7*G*,*K*,*L*).

## Discussion

Here, we applied scRNA‐seq technology to understand the process of human and murine osteoclastogenesis and found that different maturation clusters coexist during in vitro osteoclast differentiation in both humans and mice. Recent studies using single‐cell analysis have revealed new origins of OCPs and osteoclasts. Yahara and colleagues^(^ ^15^ ^)^ investigated the origins of OCPs using a cell fate‐mapping study and scRNA‐seq analyses, which revealed a population of erythromyeloid progenitor‐derived OCPs that differentiate into osteoclasts. Yu and colleagues^(^ ^14^ ^)^ found adipogenic lineage precursors in bone marrow by performing scRNA‐seq and showed that the precursors could promote OCPs into osteoclasts by providing RANKL. Tsukasaki and colleagues^(^ ^16^ ^)^ examined osteoclasts and their precursors in vitro and found that the precursors transiently expressed *CD11c*; they also identified the *Cited2* gene as the molecular switch that triggered osteoclast differentiation. Our data have shown that there are more than 10 clusters in human and murine osteoclast cultures. Using integrated single‐cell data from humans and mice and trajectory analysis, we have revealed that OLCs in the mitosis (G2/M) phase do not differentiate into mature osteoclasts. The majority of the mature osteoclasts were derived from OLCs in G1/S phase. Most of the human OLCs or preosteoclasts were in G1 phase, whereas the murine OLCs were in G1/S phase. The differences in the cell‐cycle phase of the clusters may lead to the distinct differences in maturation of OLCs between humans and mice.

Our integrated data also demonstrated the critical role played by *RAB38* in osteoclast activation. Rab family members belong to the Ras GTPase superfamily and are involved in membrane trafficking.^(^ ^34^ ^)^ Rab proteins are ubiquitously expressed, with more than 60 Rab family members having been identified in mammalian cells.^(^ ^35^ ^)^ Several previous studies have reported the functions of Rab family members in osteoclasts. Rab3 regulates the formation of the ruffled membrane found in osteoclasts,^(^ ^32^ ^)^ whereas Rab7 is distributed around the nuclei and translocates into the fusion zone of the ruffled border in resorbing rat osteoclasts.^(^ ^33^ ^)^ However, our scRNA‐seq data failed to show the expression of Rab3 or Rab7 in human or murine OLCs or mature osteoclasts (Figs. S4 and S5).

The analysis of osteoclasts generated from Rab38 knockout mice has previously been reported. Charles and colleagues^(^ ^36^ ^)^ screened Nfatc1‐dependent transcripts by analyzing the gene expression profiles of Nfatc1‐deficient osteoclasts and identified Rab38 as an Nfatc1‐dependent molecule. However, Rab38 knockout mice did not show any differences in skeletal phenotypes compared with wild‐type mice. It has been reported that Rab38 collaborates with Rab32 in the trafficking of melanogenic enzymes^(^ ^37^ ^)^ and that the combined deficiency of Rab38 and Rab32 impairs thrombosis in mice.^(^ ^38^ ^)^ Therefore, the lack of an in vivo bone phenotype in Rab38 knockout mice may be due to the redundancy of Rab family members. Our results showed that in vitro gene silencing of Rab38 by Cas9/gRNA tended to increase the expression of some Rab family genes, such as Rab12, Rab18, and Rab34. It may suggest the existence of a complementary mechanism in the Rab family for the cytoskeletal organization. The skeletal phenotype of osteoclast‐specific Rab38‐deficient mice should be analyzed to understand in further detail the physiologic role Rab38 plays in osteoclasts.

This study has some limitations. First, we used different origins of OCPs in humans and mice as there were some technical issues to obtain the tissues and cells. Human osteoclasts were differentiated from PBMCs‐derived macrophages, whereas murine osteoclasts were originated from BMMs. These different origins of cells would contain other immature stem cells. It is also reported that the difference of response to cytokines such as TGF‐β in PBMC‐derived and BMM‐derived OCPs affects the progression of osteoclastogenesis.^(^ ^39^ ^)^ These factors may influence the results of this study. Additional experiments would be necessary to elucidate the differences and the underlying mechanisms. Second, the mature osteoclasts were too large for scRNA‐seq analysis. Osteoclasts are multinucleated giant cells, with mature osteoclasts exceeding 100 μm in diameter. The chip diameter of the microfluidic channel used in the scRNA‐seq assay using 10× Genomics is just 50 to 60 μm; therefore, we may have lost very large cells during the analysis. Besides, we collected osteoclasts from culture plates. However, osteoclasts firmly attach to culture plates, and the detachment of the cells from plates could change the cells' phenotype and gene expression. Last, to integrate data sets from different species, we reconstructed data by extracting common homologs from the human and murine data sets. Therefore, any homologs they did not share in common were omitted from the integrated analyses.

In summary, we succeeded for the first time in obtaining scRNA‐seq data from human and murine osteoclast cultures and analyzing the similarities and differences between cells from these two species at the level of single‐cell resolution. Identifying essential factors across species by analyzing integrated single‐cell data will be a valuable tool for identifying therapeutic targets in bone diseases.

## Author Contributions

**Yasunori Omata:** Conceptualization; data curation; formal analysis; funding acquisition; investigation; methodology; project administration; resources; validation; visualization; writing – original draft; writing – review and editing. **Hiroyuki Okada:** Formal analysis; investigation; methodology; software; validation; visualization. **Steffen Uebe:** Investigation; methodology; software; validation. **Naohiro Izawa:** Data curation; investigation; validation; visualization. **Arif B. Ekici:** Methodology; software; supervision; validation. **Kerstin Sarter:** Funding acquisition; investigation; resources; validation. **Taku Saito:** Funding acquisition; investigation; project administration; supervision. **Georg Schett:** Funding acquisition; project administration; supervision; writing – original draft. **Sakae Tanaka:** Funding acquisition; project administration; supervision; writing – original draft. **Mario M. Zaiss:** Funding acquisition; project administration; supervision; writing – original draft.

## Conflicts of Interest

The authors declare no competing interests.

### Peer Review

The peer review history for this article is available at https://publons.com/publon/10.1002/jbm4.10631.

## Supporting information


**Fig. S1** Human and murine osteoclast scRNA‐seq processing. General methodological information for the analysis of scRNA‐seq data (*A*) and the processing of integrated human and murine scRNA‐seq data (*B*).
**Fig. S2.** Processing and analyses of the human osteoclast scRNA‐seq data set. For processing scRNA‐seq data, the dimensions and the resolution were determined using the following method. An elbow plot of the standard deviation in each principal component after preprocessing (*A*). The proportion of expressed mitochondrial genes in the total genes on UMAP (*B*). Clustree illustrating the division of cells among clusters (*C*). Dot plots (*D*) and feature plots of the expression of RAB family genes (*E*). Top 10 enriched gene ontology (GO) terms in each cluster (*F*).
**Fig. S3.** Processing and analyses of the murine osteoclast scRNA‐seq data set. For processing scRNA‐seq data, the dimensions and the resolution were determined using the following method. An elbow plot of the standard deviation in each principal component after preprocessing (*A*). The proportion of expressed mitochondrial genes in the total genes on UMAP (*B*). Clustree illustrating the division of cells among clusters (*C*). Dot plots (*D*) and feature plots of the expression of RAB family genes (*E*). Top 10 enriched GO terms in each cluster (*F*).
**Fig. S4.** Processing and analyses of the human and humanized mouse osteoclast scRNA‐seq data set. For processing scRNA‐seq data, the dimensions and the resolution were determined using the following method. An elbow plot of the standard deviation in each principal component after preprocessing (*A*). Proportion of expressed mitochondrial genes in the total genes on UMAP (*B*). Clustree illustrating the division of cells among clusters (*C*). Dot plots (*D*) and feature plots of the expression of RAB family genes (*E*). Top 10 enriched GO terms in each cluster (*F*).
**Fig. S5.** Processing and analyses of the murinized human and murine osteoclast scRNA‐seq data set. For processing scRNA‐seq data, the dimensions and the resolution were determined by using the following method. An elbow plot of the standard deviation in each principal component after preprocessing (*A*). Proportion of expressed mitochondrial genes in the total genes on UMAP (*B*). Clustree illustrating the division of cells among clusters (*C*). Dot plots (*D*) and feature plots of the expression of RAB family genes (*E*). Top 10 enriched GO terms in each cluster (*F*).
**Fig. S6.** Ligand–receptor analysis in human and murine osteoclastogenesis. Relationships between ligands expressed in all clusters in each setting and their receptors expressed in focused clusters, including clusters 4 to 6, were calculated using NicheNet, considering downstream intracellular signaling and gene expression. In the rows of each heatmap, ligands expressed by cells of all clusters in each setting are listed. In the columns of each heatmap, receptors of a focused cluster are listed. The strength of each ligand–receptor relationship is illustrated using a color scale.
**Fig. S7.** Marker molecules in each cluster. Dot plot of highly expressed genes, including the clusters of differentiation (CD) molecules, chemokines, and those receptors extracted from DEGs picked up using “FindAllMarkers” in Seurat. Figures in the upper row denote up‐regulated genes in each cluster. The larger the dot, the higher the gene expression in a cluster compared with the other clusters. In contrast, figures in the lower row show down‐regulated genes in each cluster. The larger the dot, the lower the gene expression in a cluster compared with the other clusters. *p* values for each level of gene expression are illustrated using a color scale.
**Fig. S8.** Expression of marker genes measured by quantitative real‐time PCR (qPCR). *A*. Relative expression of osteoclast markers, Nfatc1 and Cathepsin K (Ctsk). *B*. Expression of Rab family genes, Rab12, Rab18, and Rab34. The values were measured in triplicate and compared against control (*p < 0.05).
