# Correction to: Interspecies Single Cell RNA Seq Analysis Reveals the Novel Trajectory of Osteoclast Differentiation and Therapeutic Targets

**DOI:** 10.1093/jbmrpl/ziae030

**Published:** 2024-03-06

This is a correction to:

Yasunori Omata, Hiroyuki Okada, Steffen Uebe, Naohiro Izawa, Arif B. Ekici, Kerstin Sarter, Taku Saito, Georg Schett, Sakae Tanaka, Mario M. Zaiss, Interspecies Single Cell RNA Seq Analysis Reveals the Novel Trajectory of Osteoclast Differentiation and Therapeutic Targets, JBMR Plus, Volume 6, Issue 7, 1 July 2022, e10631, https://doi.org/10.1002/jbm4.10631

In the originally published version of this manuscript, The following Data availability statement was omitted:

Data availability: Sequencing data of scRNAseq was deposited in DDBJ (DRA014931, DRA014917), and is publicly available.

These details have been corrected only in this correction notice to preserve the published version of record.

