# Supplementary material for: Interspecies Single‐Cell RNA‐Seq Analysis Reveals the Novel Trajectory of Osteoclast Differentiation and Therapeutic Targets
Source: JBMR Plus. 2022 May 16;6(7):e10631. doi: 10.1002/jbm4.10631 (PMC9289986; doi:10.1002/jbm4.10631)

Supplemental figure 1. Human and murine OC scRNAseq data preprocessing.

A. Scheme of scRNAseq analysis of human and murine osteoclasts.

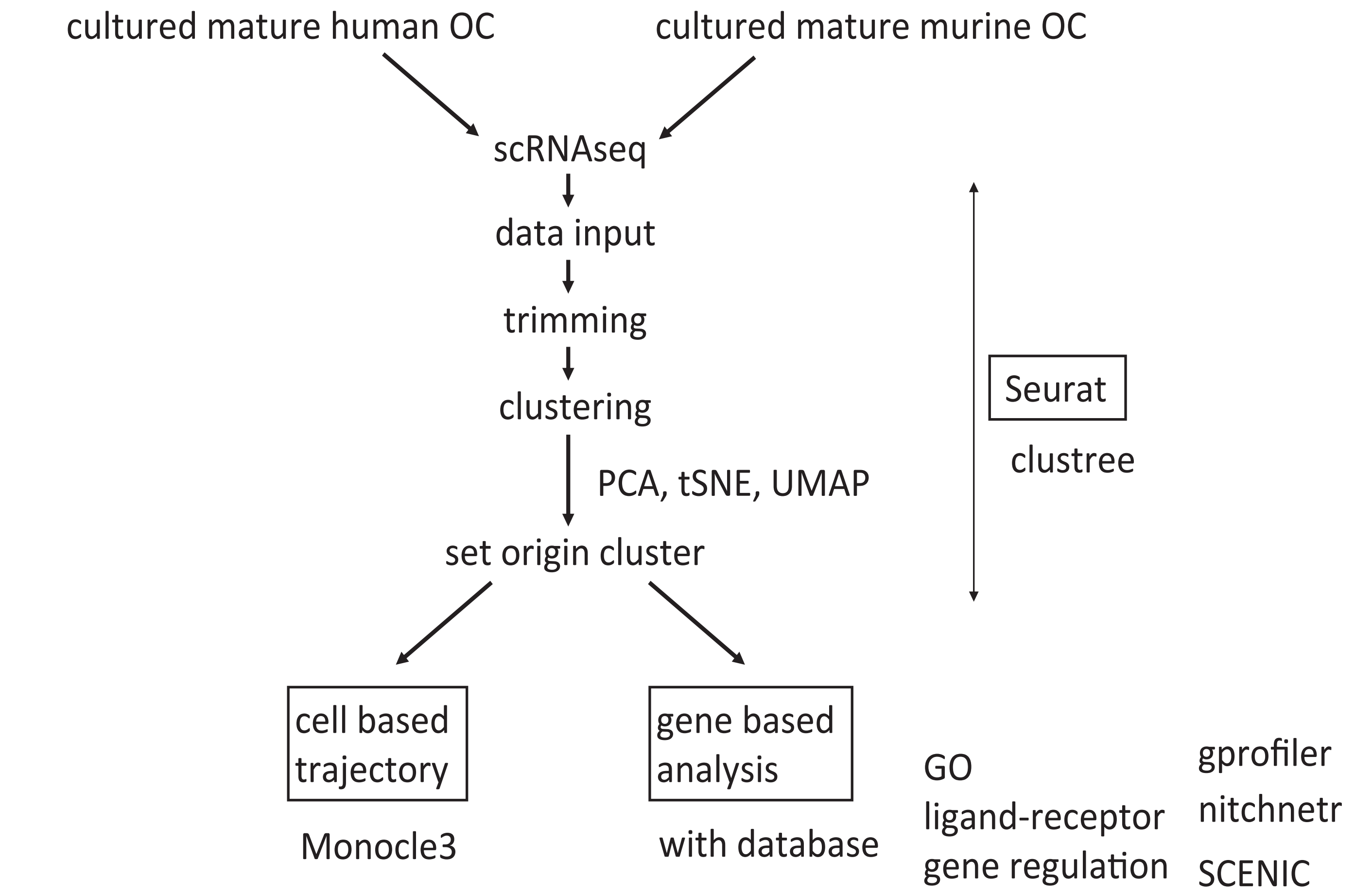

B. Scheme of merging human and murine osteoclast scRNAseq data.

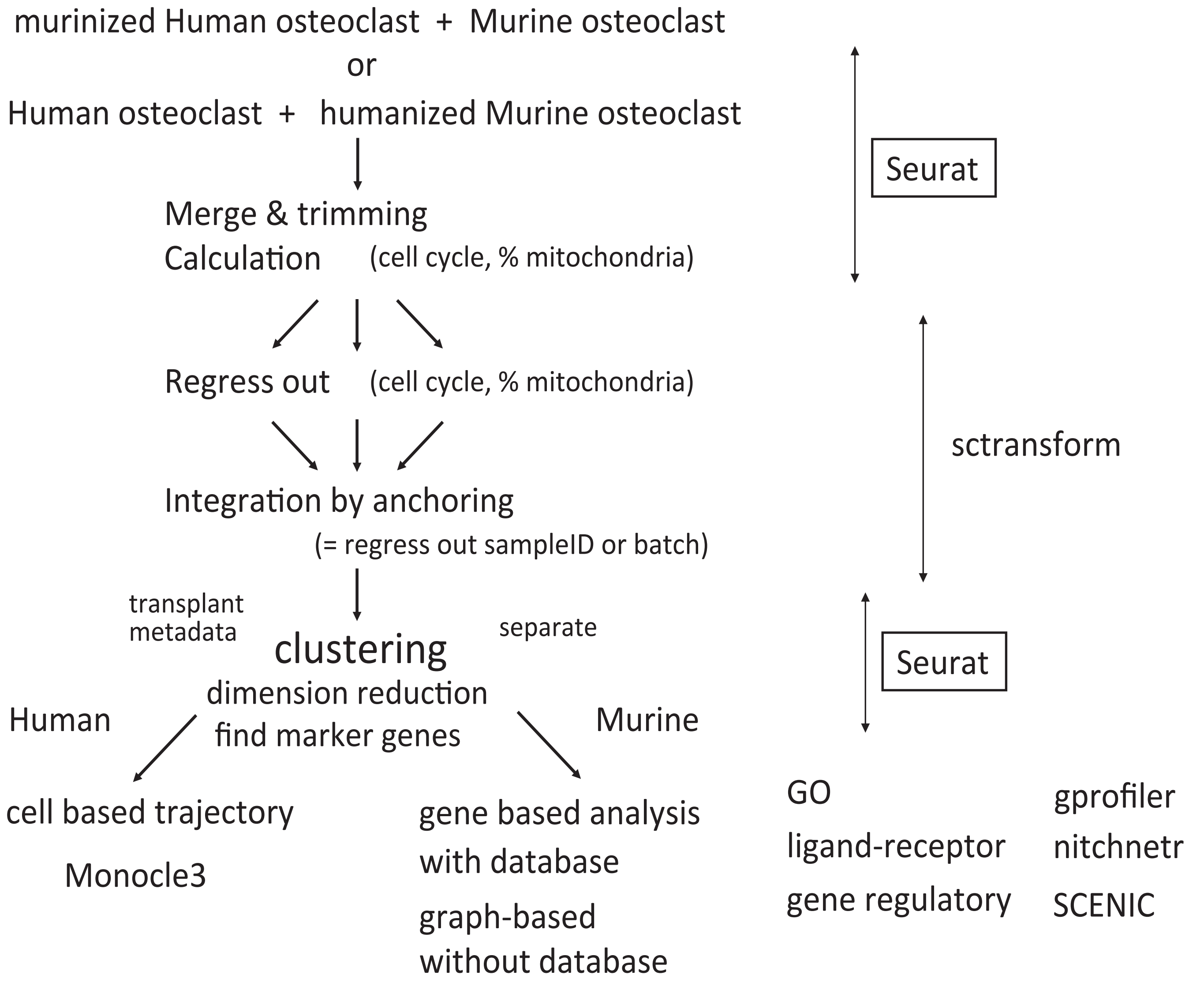

Supplemental figure.2 Human osteoclast scRNAseq data preprocessing.

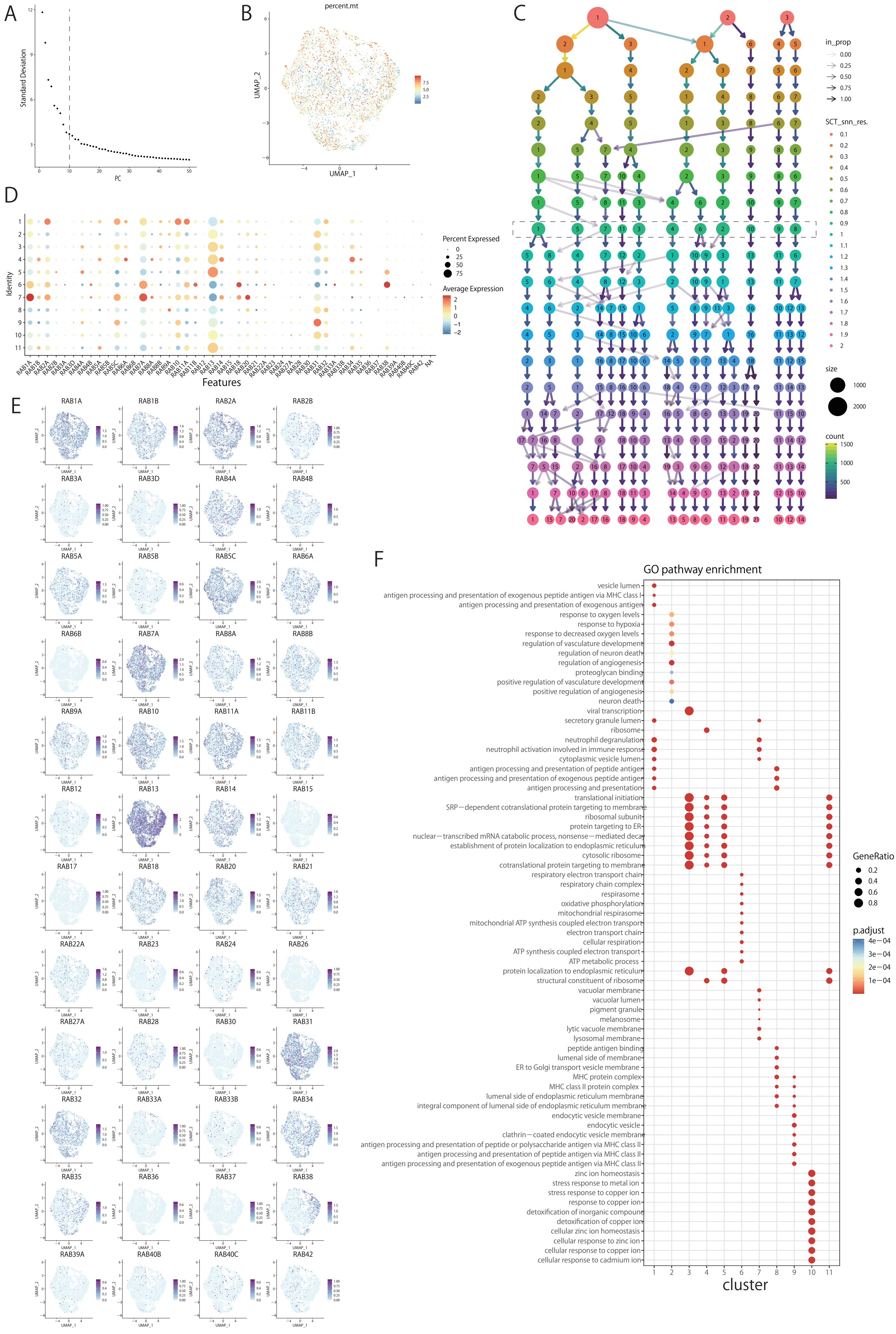

Supplemental figure.3 Murine osteoclast scRNAseq data preprocessing.

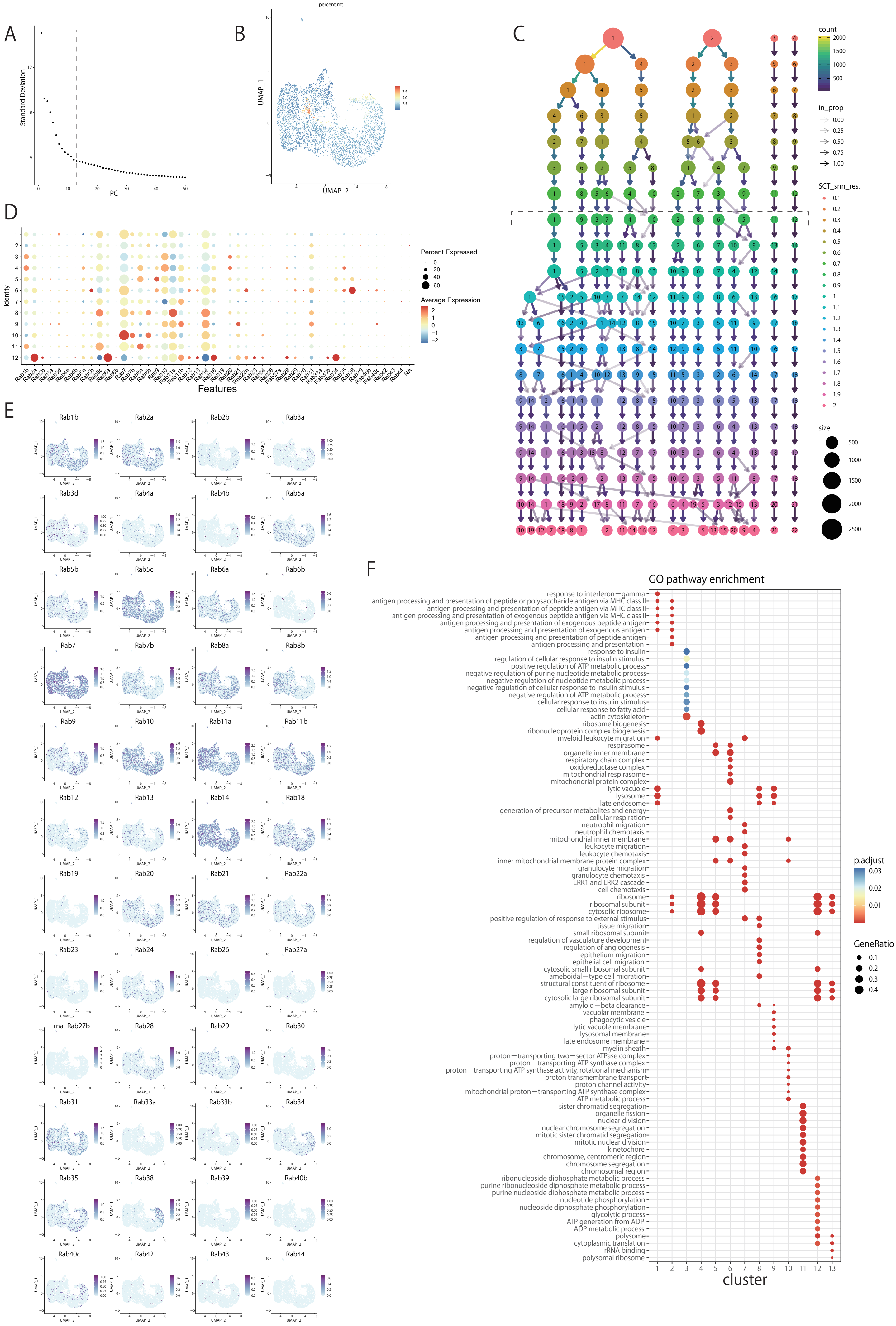

Supplemental figure.4 Human and Humanized Mouse osteoclast scRNAseq data preprocessing.

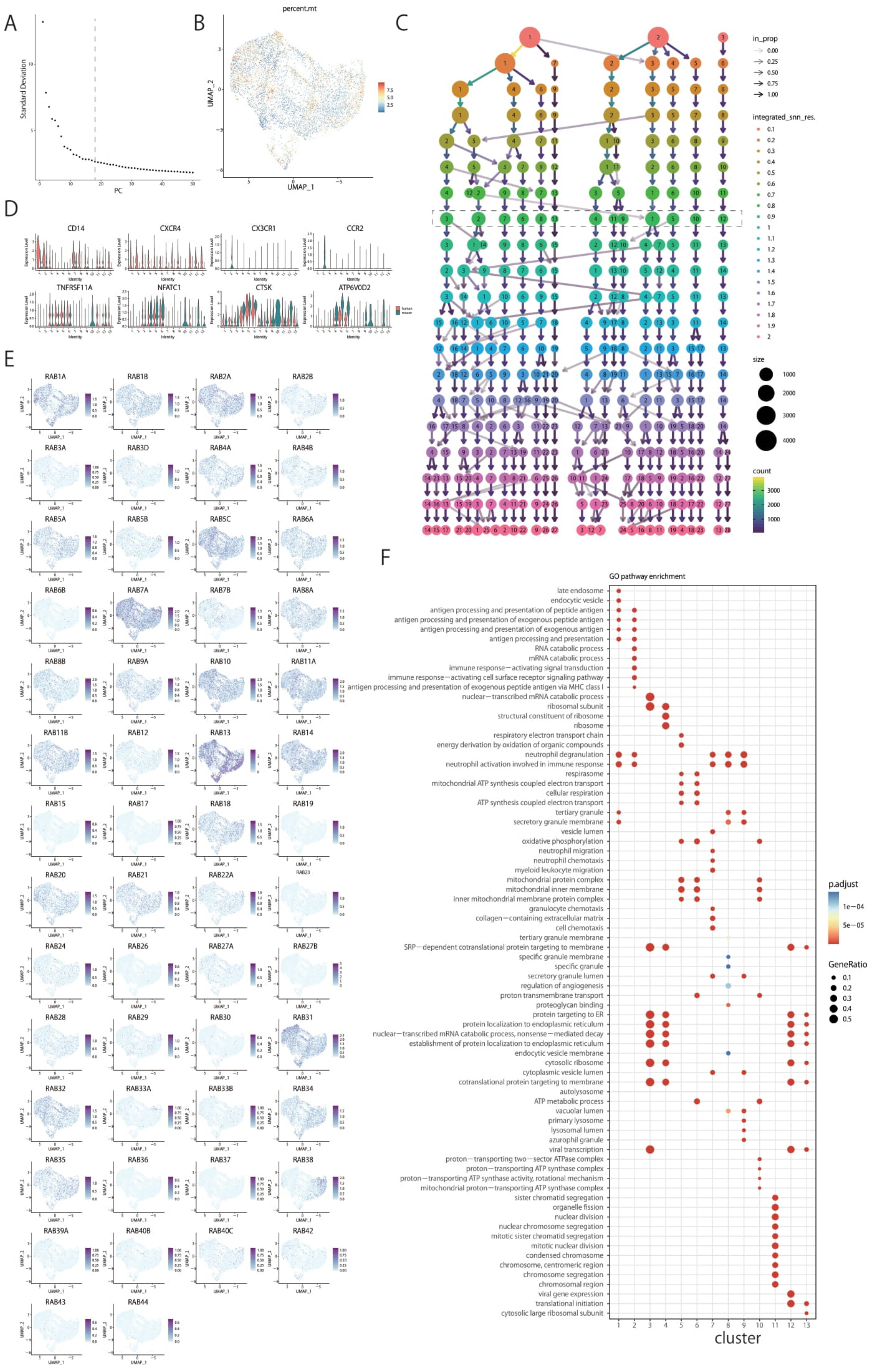

Supplemental figure.5 Murinized Human and Mouse osteoclast scRNAseq data preprocessing.

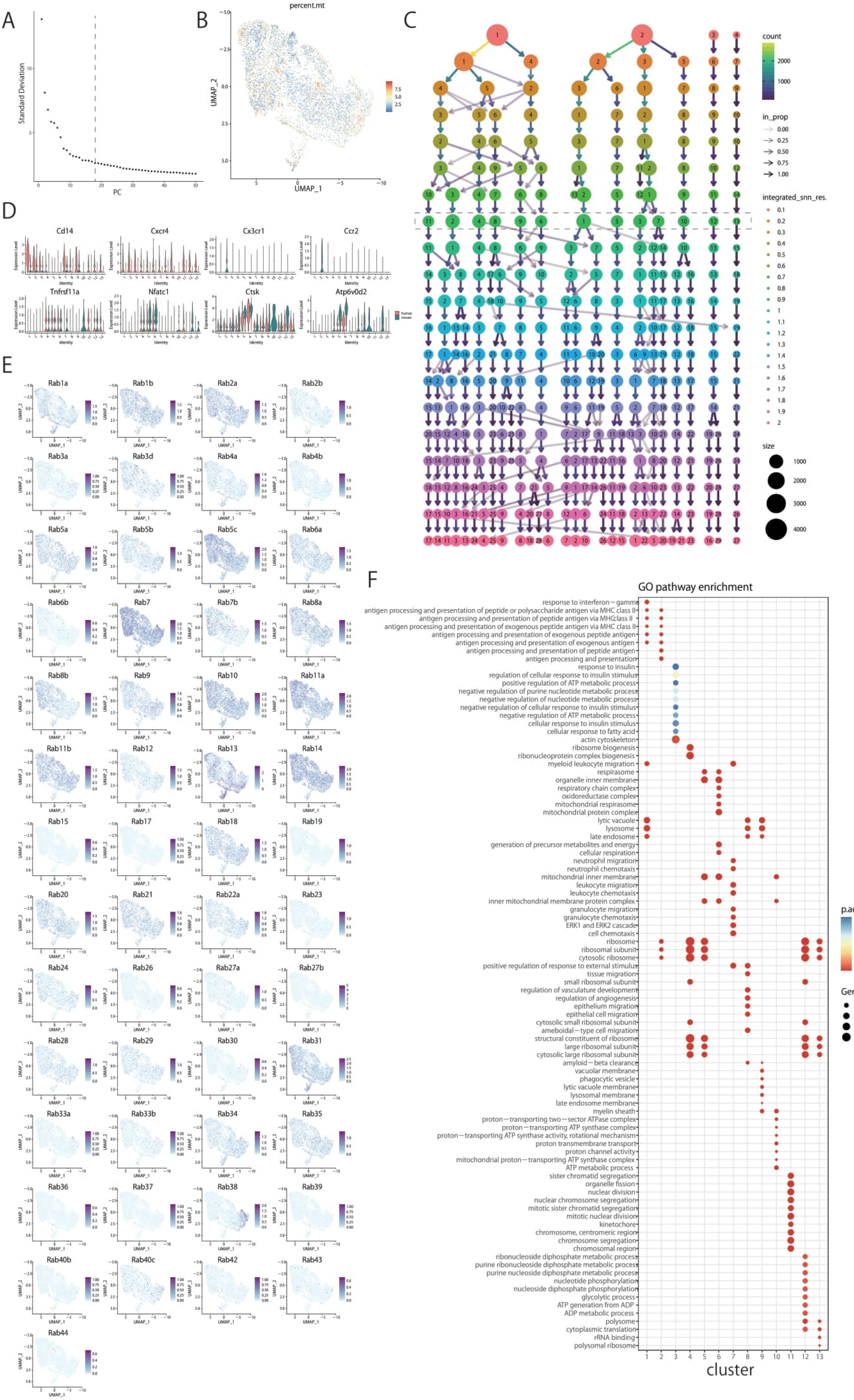

Supplemental figure.6 Ligand-receptor analysis in human and murine osteoclastogenesis.

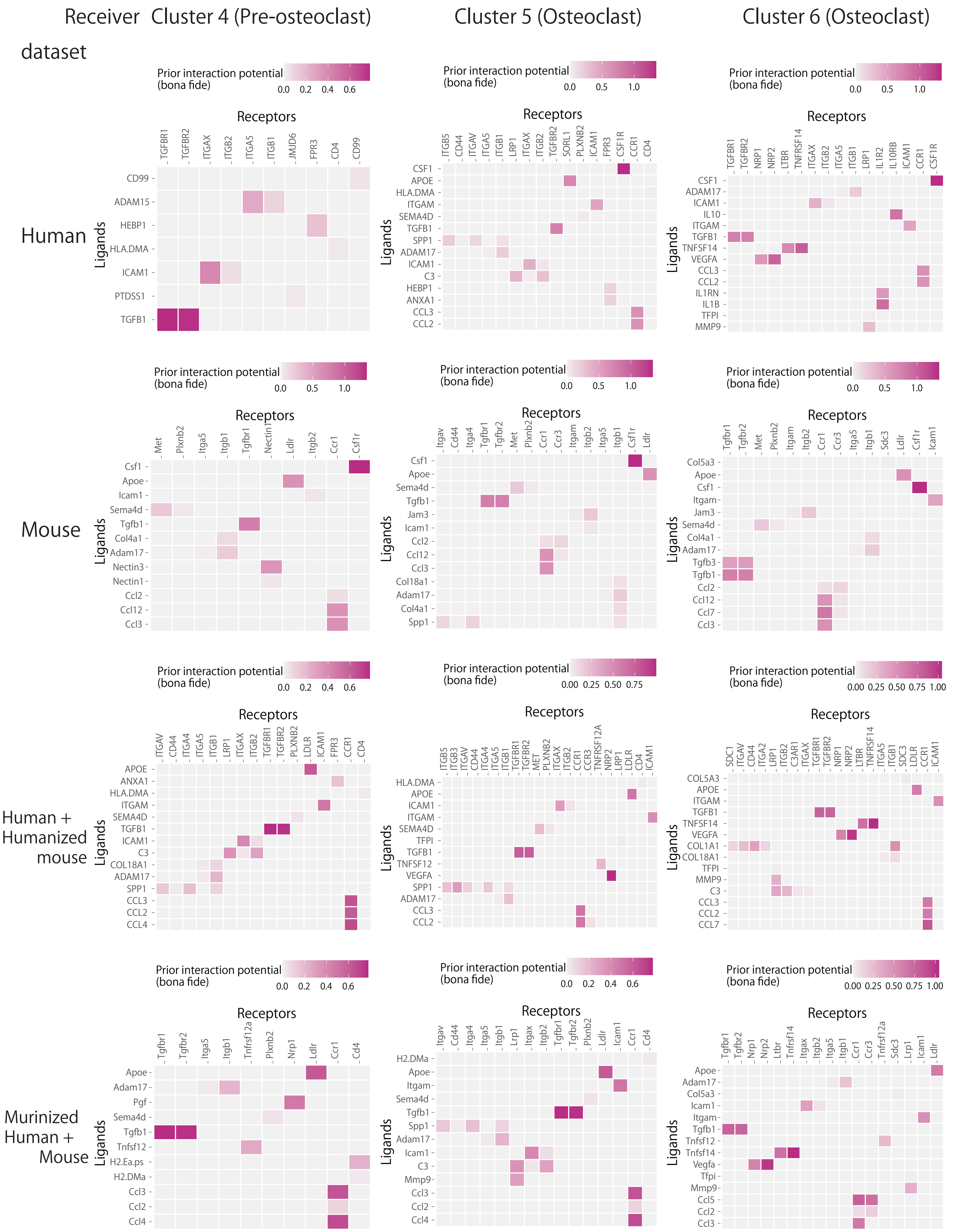

Supplemental figure.7 Marker molecules in each cluster.

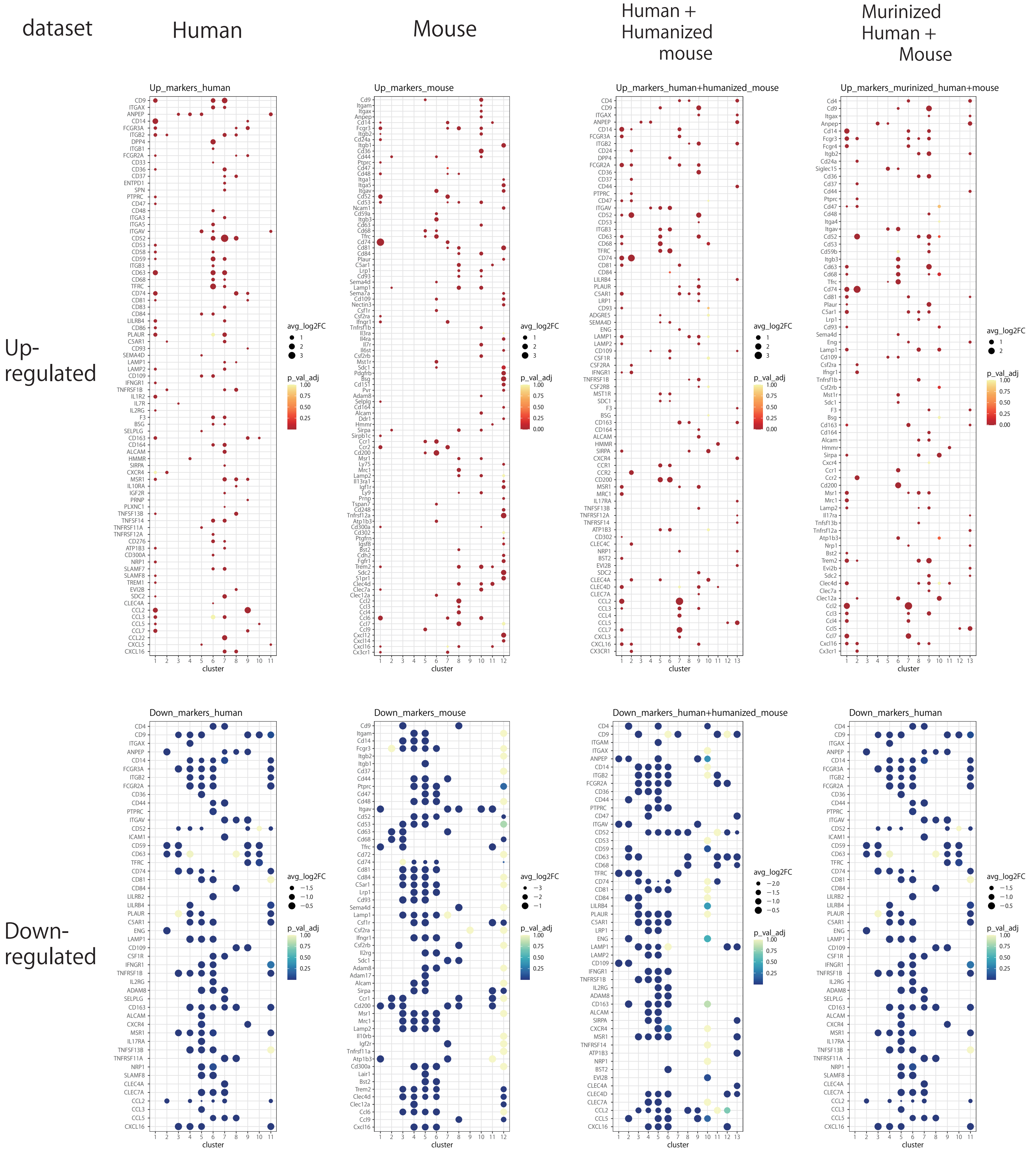

Supplemental figure. 8 Expression analaysis by qPCR.

A

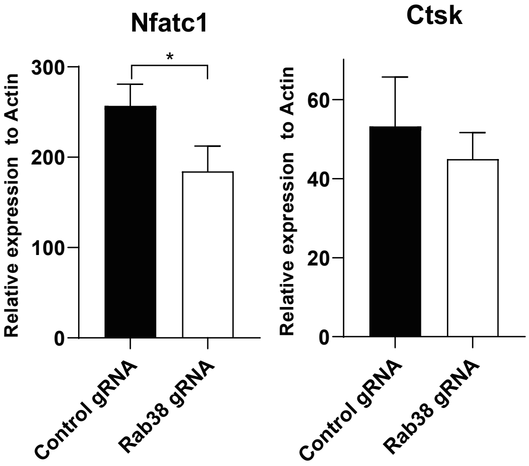

B

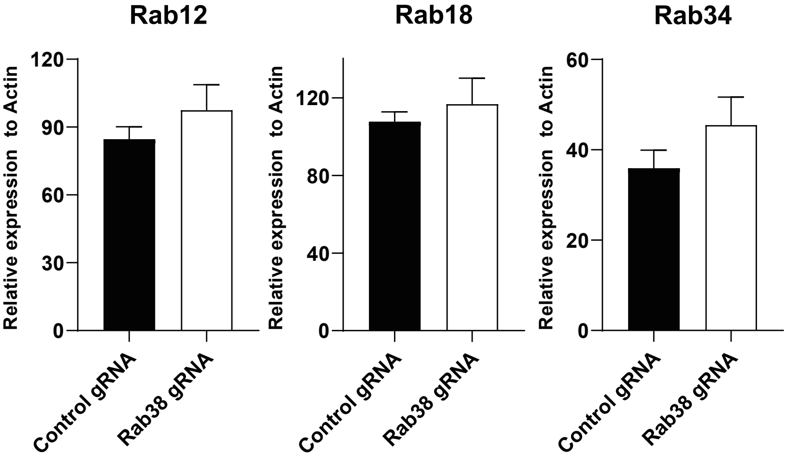

Supplement: Supplementary file 1 — Fig. S1 Human and murine osteoclast scRNA‐seq processing. General methodological information for the analysis of scRNA‐seq data (A) and the processing of integrated human and murine scRNA‐seq data (B). Fig. S2. Processing and analyses of the human osteoclast scRNA‐seq data set. For processing scRNA‐seq data, the dimensions and the resolution were determined using the following method. An elbow plot of the standard deviation in each principal component after preprocessing (A). The proportion of expressed mitochondrial genes in the total genes on UMAP (B). Clustree illustrating the division of cells among clusters (C). Dot plots (D) and feature plots of the expression of RAB family genes (E). Top 10 enriched gene ontology (GO) terms in each cluster (F). Fig. S3. Processing and analyses of the murine osteoclast scRNA‐seq data set. For processing scRNA‐seq data, the dimensions and the resolution were determined using the following method. An elbow plot of the standard deviation in each principal component after preprocessing (A). The proportion of expressed mitochondrial genes in the total genes on UMAP (B). Clustree illustrating the division of cells among clusters (C). Dot plots (D) and feature plots of the expression of RAB family genes (E). Top 10 enriched GO terms in each cluster (F). Fig. S4. Processing and analyses of the human and humanized mouse osteoclast scRNA‐seq data set. For processing scRNA‐seq data, the dimensions and the resolution were determined using the following method. An elbow plot of the standard deviation in each principal component after preprocessing (A). Proportion of expressed mitochondrial genes in the total genes on UMAP (B). Clustree illustrating the division of cells among clusters (C). Dot plots (D) and feature plots of the expression of RAB family genes (E). Top 10 enriched GO terms in each cluster (F). Fig. S5. Processing and analyses of the murinized human and murine osteoclast scRNA‐seq data set. For processing scRNA‐seq data, the [file JBM4-6-e10631-s001.pdf]
